# Using search queries for malaria surveillance, Thailand

**DOI:** 10.1186/1475-2875-12-390

**Published:** 2013-11-04

**Authors:** Alex J Ocampo, Rumi Chunara, John S Brownstein

**Affiliations:** 1 Harvard Medical School, 25 Shattuck Street, Boston 02115, MA, USA; 2 Boston Children’s Hospital, Children’s Hospital Informatics Program, 1 Autumn St, Boston 02115, MA, USA

**Keywords:** Epidemiology, Surveillance, Search query, Malaria, Thailand

## Abstract

**Background:**

Internet search query trends have been shown to correlate with incidence trends for select infectious diseases and countries. Herein, the first use of Google search queries for malaria surveillance is investigated. The research focuses on Thailand where real-time malaria surveillance is crucial as malaria is re-emerging and developing resistance to pharmaceuticals in the region.

**Methods:**

Official Thai malaria case data was acquired from the World Health Organization (WHO) from 2005 to 2009. Using Google correlate, an openly available online tool, and by surveying Thai physicians, search queries potentially related to malaria prevalence were identified. Four linear regression models were built from different sub-sets of malaria-related queries to be used in future predictions. The models’ accuracies were evaluated by their ability to predict the malaria outbreak in 2009, their correlation with the entire available malaria case data, and by Akaike information criterion (AIC).

**Results:**

Each model captured the bulk of the variability in officially reported malaria incidence. Correlation in the validation set ranged from 0.75 to 0.92 and AIC values ranged from 808 to 586 for the models. While models using malaria-related and general health terms were successful, one model using only microscopy-related terms obtained equally high correlations to malaria case data trends. The model built strictly of queries provided by Thai physicians was the only one that consistently captured the well-documented second seasonal malaria peak in Thailand.

**Conclusions:**

Models built from Google search queries were able to adequately estimate malaria activity trends in Thailand, from 2005–2010, according to official malaria case counts reported by WHO. While presenting their own limitations, these search queries may be valid real-time indicators of malaria incidence in the population, as correlations were on par with those of related studies for other infectious diseases. Additionally, this methodology provides a cost-effective description of malaria prevalence that can act as a complement to traditional public health surveillance. This and future studies will continue to identify ways to leverage web-based data to improve public health.

## Background

While malaria’s infectious cycle as well as effective treatment and prevention measures are well understood, challenges in timeliness and accuracy of surveillance persist [1,2]. Consequently, malaria remains one of the most endemic infectious diseases in the world and eradication unreachable [1,3]. In 2010, 99 countries reported on-going malaria transmission, with an estimated incidence of 216 million malaria cases and one million deaths from the disease [1]. Twenty-eight million of these cases occurred in Southeast Asia [3]. Improving surveillance may contribute to decreased malaria incidence through informing policymakers, donors and researchers on where and when to best allocate their resources. Traditional malaria surveillance systems harness data regarding hospitalizations, blood smear slide examination positivity rates and surveying. However, these systems suffer from delays associated with aggregation, information collection and bureaucracy resulting in a lack of timeliness for ensuing interventions. Also, relying on this traditional surveillance only captures a fraction of cases at it fails to capture malaria cases outside the hospital, such as home treatment or those seeking nonconventional treatments such as herbals. Mathematical models using climate and ecological data, another commonly explored method of surveillance, can predict malaria outbreaks; however, these models rely on continuous, detailed and rapid availability of certain data types [4].

Internet search query data has recently shown promise as a passive surveillance method for specific and sensitive monitoring of infectious disease activity, harnessing data that is easily processed, aggregated and visualized in near real-time [5]. Additionally, this information can be used to identify outbreaks earlier than traditional methods and fill gaps in surveillance data by enabling monitoring of early signs of health-seeking behavior, discerning information from individuals who do not seek formal medical attention as well as garnering high-resolution information [6-15]. Google Flu Trends, developed in 2009, is a validated surveillance method for monitoring influenza, currently describing influenza levels in 28 countries. By aggregating information on influenza-related Google searches, investigators correlated Google searches with existing case data from the Centers for Disease Control [16]. Noting the existence of other preventable diseases that lack vaccines and also result in high morbidity internationally, Google Dengue Trends was created in 2011. Dengue-related search query surveillance now occurs in nine countries and for some countries, at the state-level across the Americas and Southeast Asia [11]. This work has demonstrated that even countries with relatively low internet penetration [17] can accumulate sufficient search queries to build surveillance models that mirror the trends of official epidemiological data for infectious diseases. Given these prior validations and the need for improved malaria surveillance, the utility of Google search query volumes for estimating malaria incidence is due for assessment.

## Methods

### Overview

The goal of this study was to determine whether query data from malaria-related Google searches can be used to predict existing malaria surveillance trends for Thailand. The association was investigated by first acquiring official malaria case data, then selecting correlated search queries, fitting the data to a prediction model, and validating the accuracy of the model. Statistical analyses were conducted using the statistical software R, version 2.10.1 (Vienna, Austria).

### Data sources

#### Thailand: country selection and data description

Thailand was selected as the model country for malaria surveillance via internet search queries because of its malaria endemicity, availability of epidemiological data online, and substantial volume of Google query data. Official malaria surveillance data from Thailand was obtained from the official World Health Organization (WHO) website for Southeast Asia [18] and included five years of reported symptomatic malaria cases aggregated nationally by month from 2005 to 2009. Data were reverse extracted from official source graphs using GetData Graph Digitizer Version 2.24 (Moscow, Russia).

### Google search query time series

All Google query data used in the analysis were made available by Google Correlate, an open-source online tool that enables the user to identify the top 100 search queries with trends most similar to either a time series data uploaded by the user or to one particular search term of interest [12]. Both methods of query investigation were used to find correlations with official case data. First, queries statistically correlated to the official Thai monthly malaria case training data from 2005 to 2008 were returned when the data was uploaded to Google Correlate. Second, Thai physicians provided ten malaria-related terms. From these a subset of queries was selected that correlated (correlation >0.6), and then those terms were re-entered into Google Correlate to discover other related terms (correlation >0.6). No information about the identities of the users who had searched these queries is retained, and IP address is used only to identify which country the user is searching from and then removed from the data.

### Model fitting

#### Univariate models

Three univariate linear models were fit to the monthly official case count time series using each of three query sub-sets described herein. Google Correlate terms associated with official data, including queries related to microscopy, formed the first set of terms (microscopy); a second set of terms was generated by Thai physicians surveyed for this purpose (physician); the third set used a previously developed automated selection algorithm to select terms (automatic). The automatic method begins by sorting queries in order from the most correlated search term to the least. Starting with the most correlated query the automated method adds the next most correlated query to the model until the prediction obtains the best fit against out-of-sample malaria case data [6]. All three models were fit to the case data by the following equation independently:

$$M=\beta_{0}+\beta_{1}S+\epsilon \;$$

where *M* is the official malaria case count, *S* is the sum of normalized sub-set of Google search query fractions selected for each of the three models, β_*0*_ is the intercept, β_*1*_ is the multiplicative coefficient, and ϵ is the error term.

### Multivariate Akaike information criterion model

Additionally, a penalized multivariate regression model was fit to *M* (stepwise model). The model began with 20 parameters, all of the health-related queries identified by Google Correlate. Then parameters were removed in a stepwise fashion and decided on the final variable sub-set using the Akaike information criterion (AIC) as the criterion [19].

### Model validation

Statistically the models’ accuracies were determined by their ability to predict the malaria outbreak in 2009, their correlation with the entire available malaria case data, and by AIC. Queries in the models were also assessed qualitatively for their perceived strength of relationship to malaria. The official case count time series *M* was split into two sets: a training set *M*_*t*_ and a validation set *M*_*v*_. The models were trained on *M*_*t*_, which spanned from 2005 to 2008. *M*_*v*_ was used only for assessing the accuracy of the models. For *M*_*v*_, the last full malaria season in the time series was used, which was 2009. Additionally a cross-validation was performed where the four training years (M_t_) and one validation year (M_v_) were varied. Trained on *M*_*t*_, the model accuracy was determined by its ability to predict *M*_*v*_ as well as the entire available malaria case data *M*. The fit of the model on each five *M*_*v*_ in the cross-validation was averaged based on models trained on the remaining four years. Additionally, lag times of up to two months were applied to the models and noted if this increased correlations. Lastly, goodness of fit values of each of the four models was compared using the AIC.

## Results

**Figure 1:**
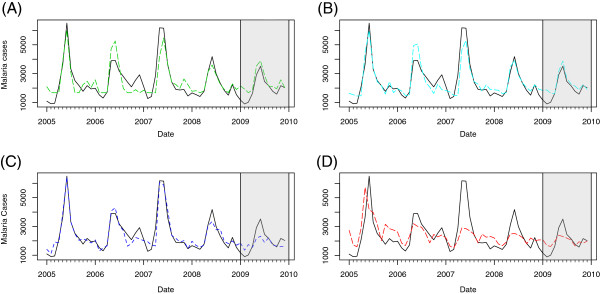
**Google search query models as compared to official case data.** Model fitted curves built on Google search queries (dashed line, colour) are juxtaposed against the official malaria case data for Thailand (solid line, black). All data shown are at a monthly resolution. Shaded regions indicate the validation year used for testing the final models. **(A)** Microscopy model: green; **(B)** Automatic model: light blue; **(C)** Stepwise model: dark blue; **(D)** Physician model: red.

The four query-based models’ were assessed statistically in their effectiveness in predicting official case data for malaria as well as on the qualitative relevancy of the queries to malaria. Each model captured the bulk of the variability in officially reported malaria incidence (Figure 1). All models appeared accurate in their ability to predict the future outbreak of 2009 as the correlation for the validation set ranged from 0.77 to 0.92 and AIC values ranged from 808 to 586. The malaria outbreak in the validation year of 2009 had fewer cases than those of previous years on which the models were built. The query fraction for the models was also smaller in this year and thus did not overestimate the outbreak, demonstrating versatility. The choice of 2009 for the validation year was not a coincidental success, as the cross-validation correlations were comparable to the validation set. These cross-validation correlations ranged from 0.78 to 0.94 (Table 1).

**Table 1:** Model correlations with official malaria case data and Akaike information criterion (AIC)

**Model**	**Overall**	**Validation set**	**Cross-validation**	**AIC**
Microscopy	0.87	0.91	0.91	760
Physician	0.60	0.77	0.78	808
Automatic	0.91	0.92	0.94	743
Stepwise	0.93	0.75	0.88	586

The physician model correlated strongly with incidence data, but was outperformed by each of the models with terms selected based on raw correlation data. Despite not fitting as well, the physician model visually appeared to be the only model that consistently captured the well-documented second seasonal malaria peak in Thailand [20]. The physician model also showed higher correlations with a one-month lag between the search query and case data. Overall, the above correlations demonstrate that search query models can represent official epidemiological trends for malaria in Thailand.

Each of the four approaches used a different number of Google queries to model official malaria case data from Thailand. The microscopy model contained three queries, the physician model contained 17, the automatic methods model contained 58 queries, and the stepwise model contained 12. Overall terms used in the models ranged from clearly relevant to malaria, to health related terms, to unrelated terms such as Buddhism. Two of the models were built completely from queries that directly related to malaria or its methods of diagnosis. These models were the microscopy and physician models. The microscopy model’s three search terms referred to microscopy, a common tool for diagnosing malaria. The terms in the physician model were hand picked or approved by Thai physicians, and thus were all directly relevant to malaria. The other two model’s terms varied in their apparent relationship to malaria. The automatic models terms included relevant and seemingly irrelevant search queries as they were selected purely by statistical correlation. The stepwise model had terms that were all health related, but not necessarily specific to malaria. A detailed description of all queries is provided in Additional file 1: Table S1.

## Discussion

Models built from Google search queries were able to adequately estimate malaria activity in Thailand, from 2005–2010, according to official malaria case counts reported by WHO. Search terms for the models were selected via correlation with case data uncovered using Google Correlate. These correlated queries were subsequently sub-setted by manual selection, an automated query selection method, and penalized multivariate regression selection. Additional queries useful for prediction were uncovered by surveying Thai physicians. This research demonstrates the potential for a method of malaria surveillance using real time Google search query data that could complement the traditional epidemiological methods in Thailand.

### Strengths and limitations

Overall, this research suggests models using Google search queries could be useful in malaria surveillance in malaria-endemic locations such as Thailand. As with other search query surveillance studies [6-15], it is not necessarily those who are sick who are online searching, but the search volume is a proxy for where there might be higher risk of disease. The inclusion of microscopy-related terms also indicates who may be searching; in this case, technicians in Thailand, using microscopes for blood smear analysis, the common malaria diagnostic method. While imperfect, these search queries may be valid real-time indicators of malaria incidence in the population. Another limitation in using internet search queries for malaria surveillance is the difference in geographic distribution of malaria and that of the query data. Internet access is more common in urban, densely populated areas while malaria is commonly found in more rural areas because the mosquito vector prefers forest dwellings and a shaded environment [21]. Finally, models built using internet search queries for surveillance should be retrained on official case data periodically to ensure that terms selected continue to relate to disease incidence [16].

### Comparison to other novel surveillance methods in the literature

In past studies relating Google search queries to infectious disease trends, the queries used to build surveillance models have been directly related to the disease of interest [6,11]. Terms that are correlated but not related are removed from the modelling. However, this is a subjective process in which researchers decide which queries are sufficiently related. The other commonly used approach to build prediction models is to define disease-related queries prior to any investigation of correlations [6,8,9,14], which was employed for the physician queries model. Both of these methods have generally resulted in terms related to symptoms of the disease, reinforcing a conceptual path between the searcher and the object of surveillance. In this study, the first approach found that terms not directly related to symptoms of the disease, but instead related to the technique in diagnosing the disease, could be related to temporal patterns of the disease. In the case of Thailand, this may be indicative of who has internet access and is involved in malaria diagnosis. Ultimately the logical relationship between the query and the disease prevalence may not need to be fully understood if the tool is continually effective in public health surveillance.

The study is unique because models were created by harnessing the publically available Google Correlate tool for finding search terms that correlate with the time series data of interest. Previous studies of infectious disease surveillance have obtained the correlated search queries via collaboration with Google, or through working with disease specialists to generate lists of queries before modelling [6,10,11,13].

Correlations from three of the models were on a par with those of previous studies, and slightly lower for one model, demonstrating the feasibility of using search query surveillance for malaria [6,7,10,11,14]. Recently, other novel internet-based active surveillance methods for malaria have been explored, however search queries provide a passively available and thus continuous and potentially earlier source of information than active surveillance methods. Surveillance methods harnessing search query data can also be used to complement traditional and other novel methods, such as mobility via cell phone data and online surveys, which have value for malaria surveillance [22,23]. To the authors knowledge this is the first study using search queries for malaria surveillance.

### Implications and future outlook

In summary, monitoring Google search queries known to correlate with malaria incidence could be useful to Thai public health practitioners for detecting epidemics in real-time. Internet-based surveillance methods are not a replacement for traditional public health surveillance and diagnostic confirmation. However, they can fill gaps in current malaria surveillance because search query data are available in near real-time while traditional surveillance data is lagged on the order of days, weeks or longer [6,24]. Future research should investigate the utility of these search queries for malaria surveillance in other malaria-endemic countries and regions. As internet access increases worldwide, search queries will become more representative. Qualitative investigations could be performed to confirm if microscopy queries are correlated to malaria incidence as the result of laboratory technicians and doctors researching malaria diagnostics or hospitals ordering more microscopy equipment during epidemics, discerning the temporal significance of these searches. Future query-based surveillance research should strongly evaluate different query selection processes as well, as these can have varied performance compared to disease incidence data. More broadly, this and future studies will continue to contribute to the evidence that web-based data can be effective as a public health tool.

## Abbreviations

AIC refers to the: Akaike information criterion; M: Stands for the official malaria case count; with Mt and Mv: Representing the training and validation data of the official malaria case count respectively; Finally, S: Refers to the sum of normalized sub-set of Google search query fractions selected for each of the three univariate models.

## Competing interests

The authors have declared that they have no competing interests.

## Authors’ contributions

AO carried out the collection of data, building of the statistical models, and evaluation of the models. RC assisted with data collection and modelling. AO and RC wrote the first draft of the paper. AO, RC and JSB collectively conceived the study. All authors read and approved the final manuscript.

## Supplementary Material

Additional file 1: Table S1 Queries used to predict malaria prevalence by model. This file lists all queries used to predict malaria prevalence for Thailand. Queries are listed by each of the four statistical models in a table format. The first table lists the queries in Thai (i e, exact queries used to build the prediction models) and the second table lists these queries translated into English by a native Thai speaker. Click here for file
