# Supplementary material for: Using search queries for malaria surveillance, Thailand
Source: Malar J. 2013 Nov 4;12:390. doi: 10.1186/1475-2875-12-390 (PMC4228243; doi:10.1186/1475-2875-12-390)
Supplement: Additional file 1: Table S1 — Queries used to predict malaria prevalence by model. This file lists all queries used to predict malaria prevalence for Thailand. Queries are listed by each of the four statistical models in a table format. The first table lists the queries in Thai (i e, exact queries used to build the prediction models) and the second table lists these queries translated into English by a native Thai speaker. [file 1475-2875-12-390-S1.pdf]

Appendix. Table A. Queries used to predict malaria prevalence by model (Thai)			
Microscopy	Physician	Automatic	Stepwise
1) ส่วนประกอบ กล้องจุลทรรศน์<br>2) ส่วนประกอบ ของ กล้องจุลทรรศน์<br>3) กล้องจุลทรรศน์ แบบ ใช้ แสง	1) ไข้มาลาเรีย<br>2) มาลาเรีย<br>3) ไข้ มาเล เรี.ย<br>4) โรค โลหิต จาง<br>5) โรค มาลาเรีย<br>6) ยุง<br>7) โลหิต จาง<br>8) ยุง ลาย<br>9) การ คาย น้ำ<br>10) ดูแล สุขภาพ<br>11) โรค ทา ลั ส ซี เมีย<br>12) กำจัด ยุง<br>13) การ ดูแล สุขภาพ<br>14) ลูกน้ำ<br>15) กัน ยุง<br>16) ไข้	1) ระบบ ผิวหนัง<br>2) ข้อมูล กับ สารสนเทศ<br>3) แทน แก รม<br>4) ส่วนประกอบ กล้องจุลทรรศน์<br>5) ประเภท ของ ห้องสมุด<br>6) องค์ประกอบ ของ ระบบ สารสนเทศ<br>7) ตรัสรู้<br>8) เสียงสระ<br>9) องค์ประกอบ ของ เทคโนโลยี<br>10) ความ สำคัญ ของ พระพุทธ ศาสนา<br>11) พระพุทธเจ้า ตรัสรู้<br>12) ห้อง รับแขก<br>13) องค์ประกอบ ของ คอม<br>14) ประเทศ และ สัญชาติ<br>15) องค์ประกอบ ของ เทคโนโลยี สารสนเทศ	1) ระบบ ผิวหนัง<br>2) การ วางแผน ดูแล สุขภาพ<br>3) โรค ระบบ หายใจ<br>4) ระบบ ย่อย อาหาร<br>5) การ เจริญ เติบโต และ พัฒนาการ<br>6) ระบบ ไหล เวียน โลหิต<br>7) ระบบ โครง กระดูก

Appendix. Table B. Queries used to predict malaria prevalence by model (English Translations)			
Microscopy	Physician	Automatic	Stepwise
1) Composition of microscope<br>2) Composition of microscope<br>3) Sunlight microscope	1) Malaria<br>2) Malaria<br>3) Malaria<br>4) Anemia<br>5) Malaria<br>6) Mosquito<br>7) Anemia<br>8) Common house mosquito<br>9) Transpiration<br>10) Health care<br>11) Thalassemia<br>12) Eradicate mosquito<br>13) Health care<br>14) Mosquito larva<br>15) Protect mosquito<br>16) Fever	1) Dermatological system<br>2) Information technology<br>3) Tangram<br>4) Composition of microscope<br>5) Type of library<br>6) Composition of information technology<br>7) Enlighten<br>8) Vowel sound<br>9) Composition of technology<br>10) The importance of Buddhism<br>11) Buddha becomes enlightened<br>12) Living room<br>13) Composition of computer<br>14) Country and nationality<br>15) Composition of information technology	1) Dermatological system<br>2) Health plan<br>3) Respiratory system<br>4) Digestive system<br>5) Growth and development<br>6) Circulatory system<br>7) Skeletal system
